# The complete chloroplast genome sequence of the chlorophycean green alga *Scenedesmus obliquus *reveals a compact gene organization and a biased distribution of genes on the two DNA strands

**DOI:** 10.1186/1471-2148-6-37

**Published:** 2006-04-25

**Authors:** Jean-Charles de Cambiaire, Christian Otis, Claude Lemieux, Monique Turmel

**Affiliations:** 1 Département de biochimie et de microbiologie, Université Laval, Québec, Canada

## Abstract

**Background:**

The phylum Chlorophyta contains the majority of the green algae and is divided into four classes. While the basal position of the Prasinophyceae is well established, the divergence order of the Ulvophyceae, Trebouxiophyceae and Chlorophyceae (UTC) remains uncertain. The five complete chloroplast DNA (cpDNA) sequences currently available for representatives of these classes display considerable variability in overall structure, gene content, gene density, intron content and gene order. Among these genomes, that of the chlorophycean green alga *Chlamydomonas reinhardtii *has retained the least ancestral features. The two single-copy regions, which are separated from one another by the large inverted repeat (IR), have similar sizes, rather than unequal sizes, and differ radically in both gene contents and gene organizations relative to the single-copy regions of prasinophyte and ulvophyte cpDNAs. To gain insights into the various changes that underwent the chloroplast genome during the evolution of chlorophycean green algae, we have sequenced the cpDNA of *Scenedesmus obliquus*, a member of a distinct chlorophycean lineage.

**Results:**

The 161,452 bp IR-containing genome of *Scenedesmus *features single-copy regions of similar sizes, encodes 96 genes, *i.e*. only two additional genes (*infA *and *rpl12*) relative to its *Chlamydomonas *homologue and contains seven group I and two group II introns. It is clearly more compact than the four UTC algal cpDNAs that have been examined so far, displays the lowest proportion of short repeats among these algae and shows a stronger bias in clustering of genes on the same DNA strand compared to *Chlamydomonas *cpDNA. Like the latter genome, *Scenedesmus *cpDNA displays only a few ancestral gene clusters. The two chlorophycean genomes share 11 gene clusters that are not found in previously sequenced trebouxiophyte and ulvophyte cpDNAs as well as a few genes that have an unusual structure; however, their single-copy regions differ considerably in gene content.

**Conclusion:**

Our results underscore the remarkable plasticity of the chlorophycean chloroplast genome. Owing to this plasticity, only a sketchy portrait could be drawn for the chloroplast genome of the last common ancestor of *Scenedesmus *and *Chlamydomonas*.

## Background

The complete chloroplast DNA (cpDNA) sequences currently available for green plants (green algae and land plants) point to radically divergent evolutionary trends of the chloroplast genome in the phyla Streptophyta and Chlorophyta. The Streptophyta [1] comprises all land plants and their closest green algal relatives, the members of the class Charophyceae sensu Mattox and Stewart [2]. In this phylum are currently available the chloroplast genome sequences of about 35 land plants and six or seven charophycean green algae (six algae if the controversial phylogenetic position of *Mesostigma viride *at the base of the Streptophyta and Chlorophyta [3-5] proves to be correct and seven algae if its association with the Streptophyta is confirmed [6-8]). The Chlorophyta [9] comprises the Prasinophyceae, Ulvophyceae, Trebouxiophyceae and Chlorophyceae. The Prasinophyceae represent the most basal divergence of the Chlorophyta [10,11] and, although the branching order of the Ulvophyceae, Trebouxiophyceae and Chlorophyceae (UTC) remains uncertain [12], chloroplast and mitochondrial genome data suggest that the Trebouxiophyceae emerged before the Ulvophyceae and Chlorophyceae [13-15]. Complete chloroplast genome sequences have been reported for five chlorophytes: the prasinophyte *Nephroselmis olivacea *[16], the trebouxiophyte *Chlorella vulgaris *[17], the ulvophytes *Oltmannsiellopsis viridis *[13] and *Pseudendoclonium akinetum *[15] and the chlorophycean green alga *Chlamydomonas reinhardtii *[18].

In nearly all photosynthetic lineages investigated thus far in the Streptophyta, the chloroplast genome harbours the same quadripartite structure and the same gene partitioning pattern, genes are densely packed and most of the genes are organized into conserved clusters [19,20], the origin of which dates back to the common ancestor of all chloroplasts [16]. The typical quadripartite structure is characterized by the presence of two copies of a large inverted repeat sequence (IR) separating a small single-copy (SSC) and a large single-copy (LSC) region. The rRNA operon always resides in the IR and is transcribed toward the SSC region. Although the IR readily expands or contracts by gaining or losing genes from the neighbouring single-copy regions [21], each of the three genomic partitions (IR, SSC and LSC) shows a distinctive and highly conserved gene content. Including 106 to 137 genes, the gene repertoire appears to have progressively shrunk from charophycean green algae to land plants [20,22]. Slight changes in intron composition of the chloroplast genome also occurred during streptophyte evolution [19,20,22]. The vast majority of introns were likely acquired early during the evolution of charophycean green algae.

In the Chlorophyta, the chloroplast genome shows extraordinary variability at the levels of its quadripartite structure, global gene organization and intron composition. The cpDNA of the prasinophyte *Nephroselmis *features the largest gene repertoire (128 genes) and the most ancestral features, including the quadripartite structure and gene partitioning pattern observed in streptophytes [16]. In contrast, all four completely sequenced UTC algal cpDNAs encode fewer genes (94–112) and are substantially rearranged [13,15,17,18]. Moreover, genes in these cpDNAs are more loosely packed than in *Nephroselmis *and most streptophyte cpDNAs, intergenic spacers usually contain short dispersed repeats (SDRs) and the coding regions of some protein-coding genes are expanded [13,15,18]. Of the four UTC cpDNAs, that of the trebouxiophyte *Chlorella *has retained the highest degree of ancestral characters; it lacks an IR but has retained many ancestral gene clusters. Both ulvophyte cpDNAs feature an atypical quadripartite structure that deviates from the ancestral type displayed by *Nephroselmis *and streptophyte cpDNAs. In each genome, one of the single-copy regions features many genes characteristic of both the ancestral SSC and LSC regions, whereas the opposite single-copy region features only genes characteristic of the ancestral LSC region. Moreover, the rRNA genes in the IR are transcribed toward the latter single-copy region. From their observations, Pombert *et al*. [13] concluded that a dozen genes were transferred from the LSC to the SSC region before or soon after emergence of the Ulvophyceae and that the transcription direction of the rRNA genes changed. In the chlorophycean green alga *Chlamydomonas*, the single-copy regions are similar in size and both their gene contents and gene organizations display tremendous differences relative to the same cpDNA regions in ulvophytes, implying that numerous genes were exchanged between opposite single-copy regions during the evolutionary period separating the Ulvophyceae and the chlorophycean clade represented by *Chlamydomonas *(a clade known as the Chlamydomonadales or CW clade [11]). Gene reshuffling was so extensive that no reliable scenario of gene rearrangements can be predicted to explain the observed differences.

To gain insights into the various changes that underwent the chloroplast genome in the Chlorophyceae, we have undertaken the complete sequencing of the chloroplast genome from distinct lineages of this class. We report here the 161,452 bp chloroplast genome sequence of *Scenedesmus obliquus*, a member of the lineage that appears to share a sister relationship with the Chlamydomonadales (Sphaeropleales or DO clade) [23,24]. All swimming cells in this lineage are biflagellates with a directly opposed (DO) arrangement of basal bodies, instead of the clockwise (CW) arrangement seen in the Chlamydomonadales. *Scenedesmus *cpDNA was found to be a compact genome that carries as many derived features as its *Chlamydomonas *homologue. It shares with *Chlamydomonas *cpDNA single-copy regions of similar sizes, an almost identical gene repertoire and several derived gene clusters; however, the sets of genes in the single-copy regions of these chlorophycean genomes are very different. These extensive differences in global gene arrangement underscore the remarkable plasticity of the chloroplast genome in the Chlorophyceae.

## Results

### General features

**Figure 1:**
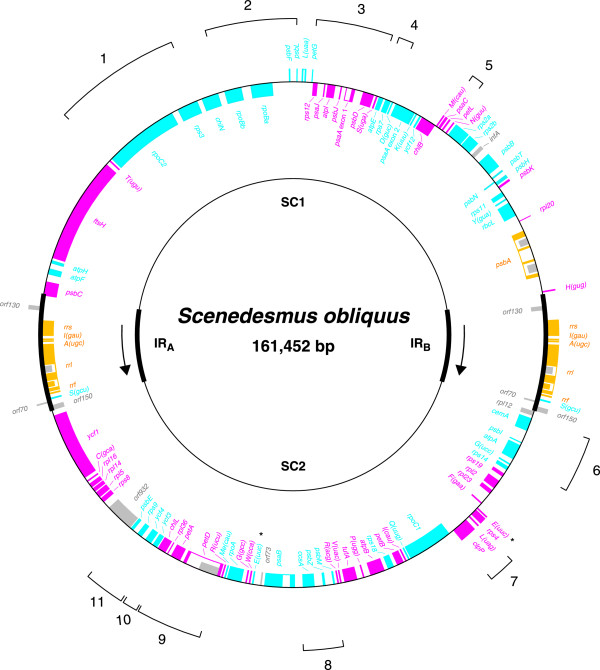
**Gene map of *Scenedesmus *cpDNA and compared patterns of gene partitioning in *Chlamydomonas *and *Scenedesmus *cpDNAs**. The two copies of the rRNA operon-containing IR (IR_A _and IR_B_) are represented by thick lines; the transcription direction of the rRNA genes is indicated by arrows. Genes (filled boxes) on the outside of the map are transcribed in a clockwise direction; those on the inside of the map are transcribed counterclockwise. The colour-code denotes the genomic regions containing the homologous genes in *Chlamydomonas *cpDNA: cyan, SC1; magenta, SC2; yellow, IR. Genes and ORFs absent from *Chlamydomonas *cpDNA are shown in grey. Labelled brackets denote the gene clusters shared specifically by *Scenedesmus *and *Chlamydomonas *cpDNAs (see Table 4 for the gene content of these clusters). tRNA genes are indicated by the one-letter amino acid code followed by the anticodon in parentheses (Me, elongator methionine: Mf, initiator methionine). Identical copies of the *trnE*(uuc) genes are denoted by asterisks. Introns are represented by open boxes and intron ORFs are denoted by narrow, filled boxes. The intron sequences bordering the *psaA *exons (*psaA *exon 1 and *psaA *exon 2) are spliced in *trans *at the RNA level. Note that only one of the two isomeric forms of the genome is shown here; these isomers differ with respect to the relative orientation of the single-copy regions.

The *Scenedesmus *cpDNA sequence assembles as a circular molecule of 161,452 bp encoding a total of 96 genes (not counting intron ORFs, free-standing ORFs and duplicated genes) (Fig. 1 and Table 1). With an overall A+T content of 73.1%, this chloroplast genome is the most A+T rich among completely sequenced chlorophyte cpDNAs. Two identical copies of an IR sequence of 12,022 bp are separated from one another by single-copy regions differing by only 7.5 kbp in size (SC1 and SC2). For both isomeric forms of the genome, a remarkably strong bias is observed in the distribution of genes between the two DNA strands. In the isomer shown in Fig. 1, 82 genes occupy one strand whereas, only 20, including the six present in the IR, reside on the opposite strand. Furthermore, 64 consecutive genes, encompassing more than half of the genome, feature the same polarity. The genes in *Scenedesmus *cpDNA are more tightly packed than in the four other completely sequenced UTC algal cpDNAs, their density (67.2%) being comparable to that found in *Nephroselmis *cpDNA (68.7%). Intergenic spacers have an average size of 465 bp and feature short dispersed repeats (SDRs). A total of nine introns, seven group I and two group II introns, were identified in *Scenedesmus *cpDNA; five of these introns display ORFs.

**Table 1:** General features of *Scenedesmus* and other UTC algal cpDNAs

**Feature**	***Chlorella***	***Oltmannsiellopsis***	***Pseudendoclonium***	***Scenedesmus***	***Chlamydomonas***
Size (bp)					
Total	150,613	151,933	195,867	161,452	203,827
IR	– ^a^	18,510	6,039	12,022	22,211
LSC	– ^a^	33,610	140,914	72,440 ^b^	81,307 ^b^
SSC	– ^a^	81,303	42,875	64,968 ^c^	78,088 ^c^
					
A+T (%)	68.4	59.5	68.5	73.1	65.5
					
Coding sequences (%) ^d^	60.9	59.2	62.3	67.2	50.1
					
Genes (no.) ^e^	112	105	105	96	94
					
Introns (no.)					
					
Group I	3	5	27	7	5
Group II	0	0	0	2	2

^a^Because *Chlorella *cpDNA lacks an IR, only the total size of this genome is given.

^b^In this study, this region is designated SC1 rather than LSC because it displays major differences in gene content relative to the LSC region in *Mesostigma *and *Nephroselmis *cpDNAs [13].

^c ^This region is designated SC2 rather than SSC because it displays major differences in gene content relative to the SSC region in *Mesostigma *and *Nephroselmis *cpDNAs [13].

^d^Conserved genes, unique ORFs and introns were considered as coding sequences.

^e^Genes present in the IR were counted only once. Unique ORFs and intron ORFs were not taken into account.

### Gene content and gene structure

The gene repertoire of the *Scenedesmus *genome differs from that of its *Chlamydomonas *homologue only by the presence of two additional genes, *infA *and *rpl12 *(Table 1). As in *Chlamydomonas *cpDNA, two identical copies of the *trnE*(uuc) gene are found on opposite strands outside the IR. Five ORFs with more than 65 codons were identified in intergenic regions (Fig. 1). The largest one, ORF932, resides in SC1 and is part of the long segment carrying genes with identical polarity. The protein encoded by this ORF shows limited sequence similarity with bacterial reverse transcriptases, the observed similarity being restricted to domain X. The four remaining ORFs display no homology with any known DNA sequences. All five ORFs differ from the conserved protein-coding genes at the levels of codon usage and nucleotide composition.

As is the case for *Chlamydomonas *cpDNA, the *rpoB *and *rps2 *genes in *Scenedesmus *cpDNA each occur as two contiguous ORFs (Fig. 1) and two separate genes, *clpP *and *rps3*, have extensions in their coding sequences that are absent from other chlorophyte and streptophyte cpDNAs (Table 2). The extra coding sequences in each of these genes share the same insertion site in the two chlorophycean algae. Note that the intein gene previously identified within the *Chlamydomonas eugametos clpP *gene [25] lies at a position different from the insertion sequence reported here for *Scenedesmus clpP*. Six additional protein-coding genes in both *Scenedesmus *and *Chlamydomonas *cpDNAs resemble their *Chlorella *and/or ulvophyte homologues in exhibiting expanded coding regions (Table 2). In sharp contrast, the rRNA and tRNA genes of UTC algae show less than 1% deviation in size relative to their homologues in *Nephroselmis *and *Mesostigma*.

**Table 2:** Expanded genes in *Scenedesmus* and other UTC algal cpDNAs

**Gene**	***Chlorella***		***Oltmannsiellopsis***		***Pseudendoclonium***		***Scenedesmus***		***Chlamydomonas***	
	
	**Size (bp)**	**Factor **^a^	**Size (bp)**	**Factor **^a^	**Size (bp)**	**Factor **^a^	**Size (bp)**	**Factor **^a^	**Size (bp)**	**Factor **^a^
*cemA *^b^	801	1.6	1059	2.2	909	1.8	1278	2.6	1503	3.1
*clpP*	606	0.9	588	0.9	597	0.9	1614	2.3	1575	2.3
*ftsH *^b^	5163	1.9	6879	2.6	7791	2.9	10998	4.1	8916	3.3
*infA *^b^	240	1.2	222	1.1	330	1.6	306	1.5	– ^c^	– ^c^
*rpoA*	837	0.9	1527	1.6	1734	1.8	1437	1.5	2213 ^d^	2.3
*rpoB*	3906	1.2	4251	1.3	6537	2.0	4896 ^e^	1.5	4967 ^e^	1.5
*rpoC1*	2511	1.3	3066	1.5	4737	2.4	4590	2.3	5739 ^e^	2.9
*rpoC2*	4689	1.3	5580	1.5	10389	2.8	7659	2.1	9363	2.5
*rps2*	804	1.2	717	1.0	777	1.1	2747 ^e^	4.0	2731 ^e^	4.0
*rps3*	696	1.1	708	1.1	690	1.1	2103	3.3	2139	3.3
*rps18 *^b^	312	1.4	237	1.1	258	1.2	567	2.6	414	1.9
*ycf1 *^b^	2460	2.0	2427	2.0	2505	2.0	7008	5.7	5988	4.9

^a ^Expansion factor relative to the corresponding *Mesostigma *gene. Each value was obtained by dividing the size of the UTC algal gene by the size of the corresponding *Mesostigma *gene.

^b ^Genes also expanded in streptophyte cpDNAs. Note that the *ftsH *gene is designated as *ycf2 *in *Chlorella, Chlamydomonas *and land plants cpDNAs.

^c ^*infA *is missing in *Chlamydomonas *cpDNA

^d ^Size derived from our unpublished sequence data. A portion of the *rpoA *region is not annotated in GenBank:NC_005353 as a result of a sequencing error introducing a frameshift mutation.

^e ^Gene occurring as two independent ORFs. The indicated size includes the spacer separating the ORFs.

### Gene partitioning and gene clustering

Our comparison of the gene complements found in the three genomic regions of *Scenedesmus *cpDNA with those observed in the *Chlamydomonas *genome reveals dramatic differences in the gene composition of the single-copy regions (Fig. 1). In each single-copy region of *Scenedesmus *cpDNA, we find numerous genes whose homologues map to the opposite single-copy region in the *Chlamydomonas *genome. Of the 43 genes displayed by *Scenedesmus *SC1 (largest single-copy region), 24 are located in the SC1 region (largest single-copy region) of *Chlamydomonas *(genes shown in cyan in Fig. 1), whereas all the others map to the alternate SC2 region (genes shown in magenta). Similarly, 19 of the 47 genes present in *Scenedesmus *SC2 reside in the SC1 region of *Chlamydomonas*, whereas all the others lie in the opposite SC2 region. Note here that the single-copy regions of both *Scenedesmus *and *Chlamydomonas *were arbitrarily designated (see Table 1) and that the genes shared by the SC1 or SC2 regions of these algae were not necessarily confined to the same single-copy region in the chloroplast genome of the last common ancestor of the two algae. Assuming that the SC1 or SC2 regions in *Scenedesmus *and *Chlamydomonas *cpDNAs are equivalent, it would be necessary to propose that the transcription direction of the rRNA operon was altered during the evolution of chlorophycean green algae concurrently with the extensive exchanges of genes that took place between the single-copy regions.

**Figure 2:**
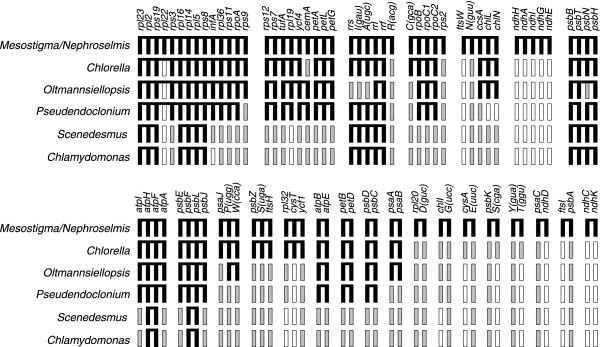
**Conservation of ancestral gene clusters in *Scenedesmus *and other UTC algal cpDNAs**. Black boxes represent the 89 genes found in the 24 clusters shared by *Mesostigma *and *Nephroselmis *cpDNAs as well as the genes in UTC algal cpDNAs that have retained the same order as those in these ancestral clusters. For each genome, the set of genes making up each of the identified clusters (either an intact or fragmented ancestral cluster) is shown as black boxes connected by a horizontal line. Black boxes that are contiguous but not linked together indicate that the corresponding genes are not adjacent on the genome. Gray boxes denote genes in UTC algal cpDNAs that have been relocated elsewhere on the chloroplast genome; open boxes denote genes that have disappeared from the chloroplast genome. Although the *rpl22 *gene is missing from *Nephroselmis *cpDNA, it is shown as belonging to the large ribosomal protein cluster equivalent to the contiguous *S10*, *spc *and α operons of *Escherichia coli *because it is present in this cluster in the cpDNAs of *Mesostigma*, streptophytes and algae from other lineages. Note also that the *psbB *cluster of *Oltmannsiellopsis *and *Pseudendoclonium *cpDNAs differs from the ancestral cluster found in other genomes by the presence of *psbN *on the alternate DNA strand.

To identify the ancestral clusters carried by *Scenedesmus *cpDNA as well as the derived clusters that are shared with other UTC algal cpDNAs, we carried out a detailed comparative analysis of gene order. As a first step, we investigated the 24 gene clusters present in both *Mesostigma *and *Nephroselmis *cpDNAs and found that *Scenedesmus *cpDNA is identical to its *Chlamydomonas *counterpart in terms of composition of ancestral clusters (Fig. 2). Both chlorophycean green algal cpDNAs harbour a single, intact ancestral cluster (*psbB-T-/N-/H*, with the slash indicating a change in gene polarity) and the remains of four other ancestral clusters: altogether, these conserved clusters encode 20 genes. These observations confirm the notion that the lowest degree of ancestral clusters among UTC algal cpDNAs is found in the chlorophycean lineage [18,26]. As previously reported by Pombert *et al*. [13], the chloroplast genome of the trebouxiophyte *Chlorella *exhibits the highest conservation of ancestral clusters among completely sequenced UTC algal cpDNAs; it has retained 11 intact clusters and four partially conserved ones that include 62 genes. The chloroplast genomes of the ulvophytes *Oltmannsiellopsis *and *Pseudendoclonium *have retained only five of the 24 ancestral clusters in an intact state as well as 13 and 10 partially conserved clusters, respectively.

The overall gene order in *Scenedesmus *cpDNA was also compared to the global gene arrangements of other completely sequenced UTC algal cpDNAs. As expected, we found that *Scenedesmus *cpDNA most closely resembles its *Chlamydomonas *counterpart at the level of derived gene clusters. The two chlorophycean genomes share 11 clusters that are not conserved in *Chlorella*, *Oltmannsiellopsis *and *Pseudendoclonium *cpDNAs (Fig. 1 and Table 4). Three of these clusters (clusters 9, 10 and 11) are adjacent on the *Scenedesmus *genome, whereas no shared derived clusters show contiguity on *Chlamydomonas *cpDNA. Only the *rpoB*-/*psbF*-*psbL *cluster originated by fusion of a fragment of ancestral cluster (*psbF*-*psbL*) with other genes. Two of the 11 derived clusters, *petA*-*petD *and *trnL*(uag)*-clpP *clusters, as well segments of the *atpA-**psbI-cemA* and *psbE-rps9-**ycf4-ycf3* clusters (relevant segments are underlined) have been previously identified not only in *C. reinhardtii *and its close relative *C. gelatinosa *[27] but also in the distantly pair of interfertile algae *C. eugametos *and *C. moewusii *[26] and their close relative *C. pitschmannii *[28], indicating their conservation in the Chlamydomonodales. The *Scenedesmus *chloroplast genome shares no specific gene clusters with *Oltmannsiellopsis *and *Pseudendoclonium *cpDNA and only one pair of genes (*psaJ-rps12*, a subset of cluster 3 in Table 4) with *Chlorella *cpDNA.

**Table 3:** Introns in *Scenedesmus* cpDNA and homologous introns at identical gene locations in other green algal cpDNAs

***Scenedesmus *introns**				**Homologous introns**	
**Designation**	**Subgroup **^a^	**ORF location **^b^	**ORF type **^c^	**Green alga **^d^**/Intron number **^e^	**Accession no.**

So.*L(uaa)*.1	IC3	–	–	*Bryopsis plumosa *(U)	[GenBank:M61159]
				*Chlorella vulgaris *(T)	[GenBank:NC_001865]
So.*psaB*.1	IA1	–	–	*Chlamydomonas moewusii *(C)	[GenBank: M90641]
So.*psbA*.1	IA1	L5	H-N-H	–	-
So.*psbA*.2	IA1	–	–	–	-
So.*psbA*.3	IA1	L5	H-N-H	*Pseudendoclonium akinetum *i6 (U)	[GenBank: AY835431]
So.*rrl*.1	IB4	L6	LAGLIDADG	*Chlamydomonas eugametos *i5 (C)	[GenBank: Z17234]
So.*rrl*.2	IA3	L6	LAGLIDADG	*Chlamydomonas reinhardtii *(C)	[GenBank: NC_005353]
				*Chlorella vulgaris *(T)	[GenBank: NC_001865]
				*Oltmannsiellopsis viridis *i3 (U)	[GenBank: DQ291132]
				*Pseudendoclonium akinetum *(U)	[GenBank: AY835431]
So.*petD*.1	IIA	DIV	RT-X-Zn	–	–
So.*psaA*.1	IIB	–	–	*Chlamydomonas reinhardtii *i2 (C)	[GenBank: NC_005353]

^a ^Group I introns were classified according to Michel and Westhof [66], whereas classification of group II introns was according to Michel *et al*. [65].

^b ^L followed by a number refers to the loop extending the base-paired region identified by the number; D refers to a domain of group II intron secondary structure.

^c ^For group I intron ORFs, the conserved motif in the predicted homing endonuclease is given; for the *petD *intron ORF, RT, X and Zn refer to the reverse transcriptase, maturase and nuclease domains of reverse transcriptases, respectively.

^d ^For the two *rrl *introns, only the homologues identified in completely sequenced genomes are given. The complete lists of introns homologous to So. *rrl*.1 and So.*rrl*.2 can be obtained in Turmel *et al*. [35] and Pombert *et al*. [13], respectively. The letter in parentheses indicates the chlorophyte lineage comprising the green alga indicated. U, Ulvophyceae; T, Trebouxiophyceae; C, Chlorophyceae.

^e ^The intron number is given when more than one intron is present.

**Table 4:** Derived gene clusters shared by *Scenedesmus* and *Chlamydomonas* cpDNAs

**Cluster no. **^a^	**Gene composition**
1	*rps3-rpoC2*
2	*rpoBb-rpoBa-/psbF-psbL*^b^
3	*psbD-psaAa-psbJ-atpI-psaJ-rps12*
4	*rps7-atpE*
5	*psaC-petL-trnN*(guu)
6	*atpA-psbI-cemA*
7	*trnL*(uag)*-clpP*
8	*ccsA-psbZ-psbM*
9	*petA-petD*
10	*chlL-rpl36*
11	*psbE-rps9-ycf4-ycf3*

^a ^Clusters are labelled as in Fig.1.

^b ^The slash indicates a change in gene polarity.

### Introns

The seven group I introns of *Scenedesmus *interrupt four genes: three introns occur in *psbA*, two in *rrl *and the two others in *psaB *and *trnL*(uaa). These introns fall within four different subgroups (IA1, IA3, IB4 and IC3), with the IA1 subgroup including the four introns present in *psaB *and *psbA *(Table 3). At 255 bp, the IC3 intron in *trnL*(uaa) is the smallest of the *Scenedesmus *introns. As homologous introns are inserted at the same position not only in the chloroplast *trnL*(uaa) genes of the chlorophytes *Bryopsis *and *Chlorella *(Table 3) but also in the *trnL*(uaa) genes of streptophytes and algae from other lineages, this intron is thought to have been inherited by vertical inheritance from the common ancestor of all chloroplasts [29]. The IA3 and IB4 introns in *Scenedesmus rrl *are also positionally and structurally homologous to previously reported introns in green plant cpDNAs (Table 3). Although the four IA1 introns revealed relatively poor sequence similarity with one another, two of these introns, So.*psaB*.1 and So.*psbA*.3, were found to be clearly homologous to introns inserted at identical gene locations in *Chlamydomonas moewusii *and *Pseudendoclonium *cpDNAs, respectively (Table 3). So. *psbA*.3 and its *Pseudendoclonium *homologue display not only similar primary sequences and secondary structures, but also similar ORFs encoding potential homing endonucleases carrying the H-N-H motif (44% identity at the protein sequence level). The two other IA1 introns of *Scenedesmus*, So.*psbA*.1 and So.*psbA*.2, represent unique insertion positions in the *psbA *gene; they are located only 5 bp and 6 bp away from the second and fourth introns in *Pseudendoclonium psbA*, respectively. For these two pairs of closely linked introns, similarity was found to be limited to the So. *psbA*.1 intron-encoded H-N-H homing endonuclease, which shares 33% sequence identity with the protein encoded by second intron in *Pseudendoclonium psbA*.

**Figure 3:**
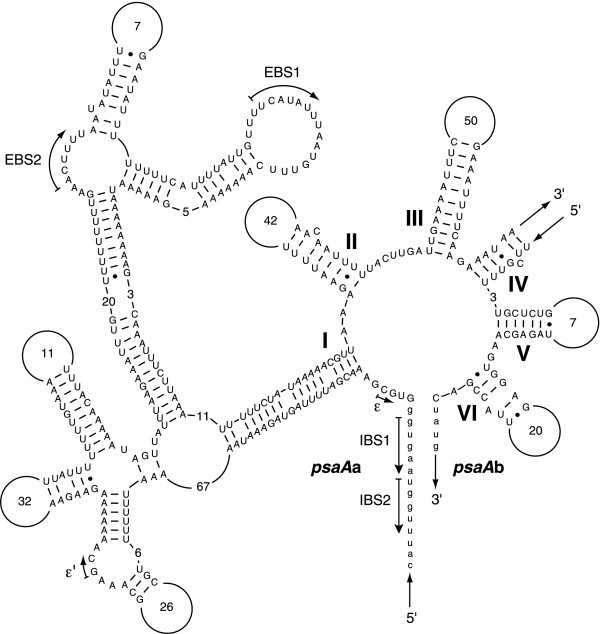
**Secondary structure model of the *Scenedesmus psaA *intron**. Intron modelling was according to the nomenclature proposed by Michel *et al*. [65]. Exon sequences are shown in lowercase letters. Roman numerals specify the six major structural domains of group II introns. Tertiary interactions are represented by blocked arrows. EBS and IBS refer to exon-binding and intron-binding sites, respectively. Numbers inside the loops denote the sizes of these regions. The 5' and 3' strand polarities of the *psaA*a and *psaA*b transcripts are indicated by arrows.

One of the two group II introns of *Scenedesmus *is spliced in *trans *at the RNA level. This intron occurs in *psaA *and is inserted at exactly the same site as the second of the two *trans*-spliced introns in *Chlamydomonas psaA *[30] (Table 3). Like its *Chlamydomonas *homologue, it has no ORF. These positionally identical *Scenedesmus *and *Chlamydomonas psaA *introns both belong to the IIB subgroup, share poor sequence similarity and are both fragmented within domain IV (Fig. 3). The second group II intron in *Scenedesmus *cpDNA lies within *petD *and has no known homologue (Table 3). This intron harbours in its domain IV an ORF encoding a reverse transcriptase [31] with the typical reverse transcriptase, maturase and nuclease domains [32].

### Short dispersed repeats

**Figure 4:**
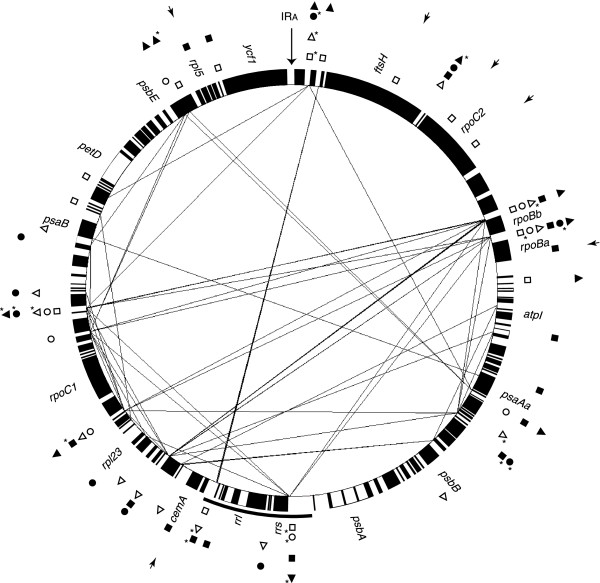
**Positions of SDR elements in *Scenedesmus *cpDNA**. Lines connect cpDNA loci displaying repeats ≥30 bp with identical sequences either on the same strand or different strands. For this analysis carried out with REPuter, one copy of the IR sequence (IR_A_) was deleted; the location of this deleted copy is indicated by the long, vertical arrow. The loci containing repeat units A, B and C are represented by symbols of different shapes outside the gene map: triangles, repeat unit A; squares, repeat unit B; circles, repeat unit C. Filled symbols denote the repeats occupying the + strand; open symbols denote the repeats found on the alternate strand. A symbol accompanied by an asterisk indicates the presence of two or more copies. Small arrows point to gene coding regions containing copies of repeat unit B.

SDR elements were identified in many intergenic spacers and some coding regions of *Scenedesmus *cpDNA; however, they are less numerous than those found in other UTC algal cpDNAs (Table 5). The repeats ≥ 30 bp in the *Scenedesmus *genome represent 8.7% of the total size of the intergenic spacers, whereas the fraction of the intergenic regions represented by such repeats in *Chlamydomonas *cpDNA reaches 31.9%. Analysis of the most abundant repeat elements in the *Scenedesmus *genome using RepeatFinder revealed three distinct groups of repeat units featuring sequences of 15 or 16 bp (Table 6). With a total of 41 copies, repeat unit B represents the most abundant group of repeats. Repeat units A and C are restricted to intergenic regions, whereas some copies of unit B are also found within the coding regions of five genes that are expanded relative to their *Mesostigma *counterparts, *i.e. cemA*, *ftsH*, *infA*, *rpoBa *and *rpoC2 *(Fig. 4 and see Table 2).

**Table 5:** Abundance of SDRs in *Scenedesmus* and other UTC algal cpDNAs

**cpDNA**		**Number of repeats **^a^		**Non-overlapping repeats ≥ 30 bp**^b^		
		
	**Maximal size of repeats (bp)**	**≥ 30 bp**	**≥ 45 bp**	**Total size (bp) **	**Fraction of genome (%)**	**Fraction of intergenic regions (%)**
*Chlorella*	84	269	44	11,743	7.8	20.8
*Oltmannsiellopsis*	172	1,205	161	18,033	11.9	30.1
*Pseudendoclonium*	171	1,047	203	10,073	5.1	13.6
*Scenedesmus*	112	86	21	4,817	3.0	8.7
*Chlamydomonas*	221	3,247	551	32,244	15.8	31.9

^a^Repeats with identical sequences on the same DNA strand or different DNA strands were identified using REPuter.

^b^The repeats identified with REPuter were annotated and masked on the genome sequence using RepeatMasker.

**Table 6:** SDR repeat units in *Scenedesmus* cpDNA

**Designation**	**Size (bp)**	**Sequence**	**Copy number **^a^	
			
			**Forward strand**	**Reverse strand**
A	15	TTTACGCTTTTTTTC	17	10
B	15	TTCTTCTTCATTTTT	22	19
C	16	TGCTTTGCTGCTTTTT	16	22

^a^Copies of each SDR unit were identified using FINDPATTERNS (one mismatch was allowed).

Although repeat units A, B and C occur on both strands of *Scenedesmus *cpDNA, they are not evenly distributed throughout the genome (Fig. 4). Many intergenic spacers entirely lack copies of these repeat units and tend to be clustered in distinct cpDNA regions (*e.g*., the regions in the vicinity of *petD*, *psbA *and *rpoC1*). On the other hand, numerous intergenic spacers are populated by two or more copies of the same repeat unit and/or by several copies representing different units (Fig. 4). The repeats in the latter spacers often form longer repeated sequences that are not randomly distributed on the *Scenedesmus *genome. In Fig. 4, it can be seen that the great majority of the repeats exceeding 30 bp in size are confined to one half of the genome. While most of the intergenic spacers harbouring SDRs reside in regions that differ in gene order relative the *Chlamydomonas *genome, some occur in shared, derived gene clusters (clusters 1, 2, 3, 5, 6 and 7).

In the intergenic spacers displaying copies of the same repeat unit, these copies are often arranged in direct orientation (Fig. 4) and separated by 23–25 bp; identical repeats also occur on different strands but, in this configuration, their distances are highly variable (8–116 bp). In intergenic regions of *Chlamydomonas *cpDNA, repeated elements appear to show arrangements similar to those reported here for *Scenedesmus *cpDNA. In contrast, in *Oltmannsiellopsis *and *Pseudendoclonium *cpDNAs, SDRs occur predominantly as stem-loop structures [13,15]. None of the repeat units of *Scenedesmus *cpDNA was identified as being part of SDRs in other UTC algal cpDNAs.

## Discussion

Our comparative analyses of the *Scenedesmus *chloroplast genome with previously sequenced chlorophyte cpDNAs highlight the remarkable plasticity displayed by the chloroplast genome in the Chlorophyceae. As expected, we found that *Scenedesmus *cpDNA shows the most similarities with *Chlamydomonas *cpDNA. The almost identical gene repertoires displayed by these chlorophycean green algal cpDNAs contrasts with the tremendous differences they exhibit at the level of gene order and pattern of gene partitioning between the single-copy regions. This highly variable gene organization is not the only surprising result that emerged from our study. Three other features of the *Scenedesmus *genome were found to be peculiar: (1) its high gene density, which mirrors that found for *Nephroselmis *cpDNA and diverges from the tendency of previously studied UTC algal cpDNAs to grow in size by gaining sequences in intergenic regions and selected gene coding regions [13,15,18], (2) the low abundance of its repeated sequences, which represents the lowest level identified thus far in a UTC algal cpDNA and (3) the strongly biased distribution of its genes between the two DNA strands.

The features shared by *Scenedesmus *and *Chlamydomonas *cpDNAs provide information on the cpDNA of the last common ancestor of DO and CW green algae; however, the portrait that could be drawn for this ancestral genome is rather sketchy owing to the major differences observed at the levels of gene order and intron content. We infer that the chloroplast genome of the last common ancestor of DO and CW green algae harboured a total of 96 genes, including a duplicated *trnE*(uuc) gene, that one third of these genes were organized in the same order as those found in the 11 gene clusters specifically shared by *Scenedesmus *and *Chlamydomonas *cpDNAs, that both *rps2 *and *rpoB *were fragmented in two pieces and that *clpP *and *rps3 *each displayed an insertion sequence. During the evolution of chlamydomonads, *infA *and *rpl12 *genes disappeared from the chloroplast genome and *rpoC1 *was broken into two separate reading frames. We also predict with confidence that the ancestral genome contained introns in *rrl *and *psaA *at the same positions as those shared by *Scenedesmus *and *Chlamydomonas *cpDNAs as well as introns in *trnL*(uaa), *psaB*, and *rrl *at the same positions as those shared by *Scenedesmus *and other chlorophyte cpDNAs. Homologues of all these introns, with the exception of the *trnL*(uaa) intron, have been identified in chlamydomonads distantly related to *C. reinhardtii *(*rrl*, [33-35]; *psaA*, [26,36]; *psaB*, [37]). In contrast, the *trnL*(uaa) intron shows a broader distribution among green algae and is thought to have been inherited from the common ancestor of all chloroplasts [29]. Undoubtedly, as reported for *C. reinhardtii *cpDNA [38], the *psaA *intron of the last common ancestor of DO and CW green algae featured a break in domain IV, the two *psaA *exons were unlinked and transcribed independently along with an intron fragment, and the intron was spliced *in trans*. In the CW lineage, a second *trans*-spliced group II intron (a tripartite intron comprising the RNA species encoded by the chloroplast *tscA *gene) took residence within *psaA *[38], group I introns inserted at several new sites within *rrl *[33,34,39] and members of the group I family also invaded multiple sites of the *rrs *[40,41], *psbA *[42] and *psbC *[37] genes.

The unusual structures displayed by the expanded *clpP *and *rps3 *genes and the fragmented *rps2 *and *rpoB *genes are inventions that arose in the Chlorophyceae. For both *clpP *and *rps3*, it has been shown that the insertion sequence is not removed at the RNA level [43,44]. Characterization of the chloroplast ClpP/R protease complex of *C. reinhardtii *revealed that the approximately 30 kDa insertion sequence in *clpP*, designated as IS1, could be a new type of intein [45]. Two distinct proteins derived from the chloroplast *clpP *gene, a long version containing IS1 and a shorter version lacking this sequence element, were found to be stable components of this complex. IS1 has been hypothesized to prevent interaction with the HSP100 chaperone and to be localized in only one of the two heptamers forming the complex, thus prohibiting access of protein substrates to the proteolytic chamber of the ClpP/R complex via one of its axial pores. In contrast, a proteomic analysis of ribosomal proteins in the small subunit of the chloroplast ribosome from *C. reinhardtii *revealed that the insertion sequence in *rps3 *is an integral part of the mature product of this gene [46]. In this same analysis, Rps2 was also found to be an unusually large ribosomal protein; this protein of 570 amino acid residues encoded by *rps2b *(the largest of the two ORFs showing sequence similarity to the bacterial rps2 genes), contains an N-terminal extension and a C-terminal half with homology to characterized Rps2 proteins from other organisms. No peptides were found to be derived from *rps2a*, indicating that the latter sequence may be a pseudogene. The biological significance of the additional domains found in Rps3 and Rps2 remains uncertain. These domains, which are exposed to the solvent side and are located near each other and around the neck of the 30S subunit, may be related to unique features of translational regulation, or they may be orthologues of nonribosomal proteins [46]. Finally, it is not yet clear how the fragmented *rpoB *gene is expressed at the protein level. This gene is undoubtedly essential for cell survival in view of the fact that, unlike their homologues in land plants, the *C. reinhardtii *nuclear genome does not appear to encode a chloroplast-targeted RNA polymerase [47,48].

The considerable differences in gene density and abundance of SDRs observed in *Scenedesmus *and *Chlamydomonas *cpDNAs raise questions about the status of the chloroplast genome of the common ancestor of DO and CW green algae with regards to these features. From the data derived from previously sequenced chlorophyte cpDNAs, Pombert *et al*. [13,15] proposed that proliferation of repeated sequences in intergenic regions and selected genes occurred progressively during the evolution of UTC algae, thereby accounting for the observation that the *Chlamydomonas *genome is the most rich in SDR elements and the least tightly packed with genes. The results reported here are compatible with this idea and support the presence of SDRs in the common ancestor of *Scenedesmus *and *Chlamydomonas *cpDNAs provided that specific loss of numerous SDRs occurred concurrently with streamlining of the genome in the *Scenedesmus *lineage. On the other hand, considering that no common SDRs have been identified in different UTC algal cpDNAs, the idea that these genetic elements were independently acquired in UTC lineages cannot be ruled out.

The single-copy regions of *Scenedesmus *and *Chlamydomonas *cpDNAs are almost equal in size but differ radically in gene content, indicating that many genes were exchanged between opposite single-copy regions during the evolution of the DO and CW algae. This observation contrasts with the situation reported for the cpDNAs of *C. reinhardtii *and *C. moewusii *[26]. These representatives of deeply branched chlamydomonad lineages also display extensive gene rearrangements in their cpDNAs; however, these rearrangements are mainly confined to individual single-copy regions. Only two (*atpA *and *psbI*) of the 77 genes mapped on the *C. reinhardtii *and *C. moewusii *genomes [26-28] moved from one single-copy region to the other.

To compare the level of gene rearrangements displayed by *Scenedesmus *and *Chlamydomonas *cpDNAs with those exhibited by other pairs of chlorophyte cpDNAs, we examined the orders of the 90 genes common to seven green algal cpDNAs (Table 7). In these analyses, changes in gene order were assumed to occur only by inversions, an hypothesis that is strongly supported by previous mapping analyses of chloroplast genes from closely related chlamydomonads [27,28]. We estimated that a minimum of 58 inversions would be required to convert the gene order of *Scenedesmus *cpDNA to that of *Chlamydomonas *cpDNA. Although extensive, this level of gene rearrangements is less important than those observed in the comparisons involving each chlorophycean genome and a genome from another green algal class (Table 7). The similar levels of gene rearrangements observed in these interclass comparisons suggest that the gene organizations of both *Chlamydomonas *and *Scenedesmus *diverged considerably from that of their last common ancestor.

**Table 7:** Minimal numbers of inversions accounting for gene rearrangements between green algal cpDNAs

**Compared cpDNA**	**Number of inversions **^a^					
	
	***Nephroselmis***	***Chlorella***	***Oltmannsiellopsis***	***Pseudendoclonium***	***Scenedesmus***	***Chlamydomonas***
*Mesostigma*	43	46	54	54	75	75
*Nephroselmis*		47	55	55	73	74
*Chlorella*			50	52	74	75
*Oltmannsiellopsis*				55	78	75
*Pseudendoclonium*					76	77
*Scenedesmus*						58

^a ^Numbers of gene permutations by inversions were computed using GRIMM.

The high gene density and strongly biased distribution of genes between the two DNA strands in the *Scenedesmus *genome most probably reflect the influence of natural selection on genome organization. A bias in clustering of adjacent genes on the same DNA strand has also been reported for the *Chlamydomonas *chloroplast genome [49]; however, this bias is less conspicuous than that observed for *Scenedesmus *cpDNA. For *Chlamydomonas *cpDNA, a parametric bootstrap approach was used to test if gene order evolves under selection [49]. In this analysis, the putative gene order in the common ancestor of *Chlamydomonas *and *Chlorella *was inferred and subjected to random rearrangements. It was found that the multiple gene rearrangements in the *Chlamydomonas *lineage resulted in an increased sidedness, *i.e*. an increased propensity of adjacent genes to be located on the same strand. Sidedness indices of 0.6966 and 0.8710 were scored for the common ancestor and *Chlamydomonas*, respectively, and simulated genomes showed a significant decrease in sidedness relative to the ancestral genome. At 0.8842, the sidedness index we calculated for *Scenedesmus *cpDNA is slightly higher than that reported for its *Chlamydomonas *counterpart.

Coding strand biases have also been reported for the plastid genomes of the parasitic green alga *Helicosporidium *sp. [50], the euglenozoan alga *Euglena gracilis *[51], and apicomplexan parasites [52-55]. This feature is prominent in the highly reduced *Helicosporidium *genome where a symmetry in strand bias of coding regions has been observed, with nearly all genes on each half of the genome being encoded on one strand. The two strands of the *Helicosporidium *and *Euglena *genomes are also biased with regards to nucleotide composition and this compositional bias switches at the putative origin of DNA replication [50,51]. It has been proposed that the coding strand bias observed in these genomes is generated by selection to code highly expressed genes on the leading strand to limit collisions between RNA and DNA polymerases, thereby increasing the rates of both replication and transcription. Unlike their *Helicosporidium *and *Euglena *homologues, *Scenedesmus *and *Chlamydomonas *cpDNAs show no strand bias in nucleotide composition (our unpublished results and [49]), thus providing no support for the notion that gene orders in chlorophycean genomes are selected to maximize the rate of replication. The high degree of sidedness observed for *Scenedesmus *and *Chlamydomonas *cpDNAs could result mainly from selection of polycistronic transcription to coordinate gene expression [49].

## Conclusion

Our study revealed that, although *Scenedesmus *and *Chlamydomonas *cpDNAs display nearly identical gene repertoires and a high level of sidedness in the distribution of their genes on the two DNA strands, their gene orders are highly scrambled. In future studies, it will be interesting to investigate whether remodelling of the chloroplast genome is subjected to different constraints in the DO and CW lineages and whether the derived state of *Scenedesmus *and *Chlamydomonas *cpDNAs arose early during the evolution of chlorophycean green algae. To test this hypothesis, it will be necessary to examine other representatives of the DO and CW clades as well as members of more basal lineages of the Chlorophyceae.

## Methods

### Strain and culture conditions

*Scenedesmus obliquus *(Turp.) Kürtz was obtained from the Culture Collection of Algae at the University of Texas at Austin (UTEX 393) and grown in modified Volvox medium [56] under 12 h light/dark cycles.

### Isolation and sequencing of cpDNA

An A+T rich fraction containing cpDNA was isolated and sequenced as described in Pombert *et al*. [14]. Sequences were edited and assembled with AUTOASSEMBLER 2.1.1 (Applied Biosystems). The fully annotated chloroplast genome sequence has been deposited in [GenBank:DQ396875].

### Analyses of genome sequence

Gene content was determined by Blast homology searches [57] against the nonredundant database of the National Center for Biotechnology and Information (NCBI) server. Protein-coding genes and open reading frames (ORFs) were localized precisely using ORFFINDER at NCBI, various programs of the GCG version 10.2 package (Accelrys, Burlington, Mass.) and other applications from the EMBOSS version 2.6.0 package [58]. Genes coding for tRNAs were localized using tRNAscan-SE 1.23 [59]. Repeated sequences were identified using REPuter 2.74 [60] and classified using REPEATFINDER [61]. Numbers of SDR units were determined with FINDPATTERNS of the GCG Wisconsin Package version 10.2. The total length of genome sequences containing repeated elements was estimated with RepeatMasker [62] running under the WU-BLAST 2.0 search engine [63].

The GRIMM web server [64] was used to infer the minimal number of gene permutations by inversions in pairwise comparisons of chloroplast genomes. Because GRIMM cannot deal with duplicated genes and requires that the compared genomes have the same gene content, genes within one of the two copies of the IR were excluded and only the genes common to all the compared genomes were analysed. The data set used in the comparative analyses reported in Table 7 contained 90 genes; the three exons of the *trans*-spliced *psaA *gene were coded as distinct fragments (for a total of 92 gene loci).

## Abbreviations

cpDNA, chloroplast DNA; CW, clockwise; DO, directly opposed; IR, inverted repeat; LSC, large single copy; SDR, short dispersed repeat; SSC, small single copy; UTC, Ulvophyceae/Trebouxiophyceae/Chlorophyceae.

## Authors' contributions

JCC participated in the conception of this study, carried out part of the genome sequencing, performed all sequence analyses, annotated the genome, generated the tables and figures, and drafted the manuscript. CO participated in the sequencing and contributed to the assembly of the genome sequence. CL and MT conceived and supervised the study, contributed to the interpretation of the data and prepared the manuscript. All authors read and approved the final manuscript.
